# Development of a Novel Rotor Design for Counter‐Current Centrifugal Extraction Based on Computational Fluid Dynamics Simulations

**DOI:** 10.1002/jssc.70339

**Published:** 2025-12-22

**Authors:** Sophia Volpert, Lisa Nordhausen, Richard Alfsmann, Gerhard Schembecker

**Affiliations:** ^1^ Laboratory of Plant and Process Design, Department of Biochemical and Chemical Engineering TU Dortmund University Dortmund Germany

**Keywords:** centrifugal partition chromatography (CPC), centrifugal partition extraction, continuous multistage extraction, hydrodynamic optimization, phase separation

## Abstract

This study introduces an innovative method for continuous multistage liquid–liquid extraction. Addressing challenges like coalescence and settling times that limit efficiency makes centrifugal partition extraction a promising technique for continuous product separation. It combines the benefits of a mixer‐settler cascade with centrifugal extractors in one compact apparatus. The working principle is based on centrifugal partition chromatography, where two immiscible liquid phases are contacted inside a rotor, one serving as the mobile phase and the other as the stationary phase. In centrifugal partition extraction, both phases are mobile. The rotating apparatus allows dispersion and reduces settling time compared with static settlers, with its 300 times higher acceleration. To overcome constructional challenges, a novel concept, termed counter‐current centrifugal extraction, is introduced in this work. Thereby, the primary objective is to validate the hypothesis that the process is feasible with a modified rotor design consisting of 10 chambers connected by individual ducts. Combined with alternating pump directions, this enables a pseudo‐continuous counter‐current flow. A proof of concept is investigated using computational fluid dynamics. The methodologies employed yield valuable insights into hydrodynamic performance. Based on these simulations, an optimized geometry for the flow area is developed, where 100% of the total chamber height is utilized as a dispersion zone compared with the previous 45%. The improvements result in elliptical chambers inclined at 35° to the radial direction, with a height of 6 mm and a volume of 1.43 mL. Additionally, baffles are placed inside the chambers opposite to the two inlets, enhancing dispersion of the inflowing phases. Finally, during alternating operation mode, it is demonstrated that ducts with a volume of 0.17 mL lead to an alternating phase ratio between 0.48 and 0.57 inside the chambers, which was repeatable over five simulated switches. This confirms the feasibility of the concept proposed based on hydrodynamics.

**Abbreviations**
CPC: centrifugal partition chromatography
CFD: computational fluid dynamics
CCE: counter‐current centrifugal extraction
CPE: centrifugal partition extraction
CPE: centrifugal partition extraction
LP: lower phase
UP: upper phase

## 1 Introduction

Increasing global competition and dynamically changing markets in the chemical and biochemical industries are leading to a greater and more complex variety of molecules and a faster production demand [1]. In order to meet these market demands, well‐known batch processes are predominantly used [1]. However, batch processes do not meet the requirements for effective, resource and energy‐efficient processes with high consistency [2, 3]. Flexible, continuous, small‐scale production plants offer the opportunity to enter future markets fast and competitively [4, 5]. Here, the downstream processing is a bottleneck in terms of costs, resource efficiency, and process times, demonstrating high potential for optimizations and novel technologies [6].

With its high flexibility in the use of solvents, liquid–liquid extraction and partitioning are very promising for downstream processes in multipurpose production. Furthermore, due to its low operating temperatures, liquid–liquid extraction is particularly suitable for temperature‐sensitive substances, which are becoming increasingly important in the biochemical industry [7, 8, 9, 10].

Especially for continuous operation, mixer‐settler cascades, or extraction columns are commonly used [11]. For efficient mass transfer during liquid–liquid extraction and, thus, time and process efficiency, dispersion and coalescence of the immiscible liquid phases are of major importance [7]. With higher degrees of dispersion, the interfacial area between the raffinate and extract phase is increased, and the mass transfer is enhanced. However, coalescence and settling are time‐consuming and needs and a lot of space, representing a major challenge to all extraction processes [7, 9, 11, 12, 13].

The more intense the dispersion, the slower the subsequent coalescence and settling of the phases. The same phenomenon underlies when the density difference between the two immiscible liquid phases is relatively small, or the viscosity is high. All these effects result in settling as a rate‐determining step or even incomplete settling combined with solvent leaching. With the focus on continuous processes, this represents a bottleneck for the entire process chain and, therefore, a major cost factor. One way to reduce settling times is to increase the mass force by applying a centrifugal field to the fluids. Hence, the demand for increased phase separation motivates performing extraction in centrifugal contactors [9, 10, 14, 15, 16].

Dependent on the size of the centrifugal extractors in combination with the rotational speed, centrifugal forces up to 6000 G can be realized, like in the annular centrifugal extractor [11]. However, conventional centrifugal extractors are limited for demanding separation tasks due to the restricted number of separation stages provided [10, 15, 17].

To overcome this challenge, schwienheer developed a promising concept for centrifugal partition extraction (CPE), patented in 2014 [18, 19]. It is based on the concept of centrifugal partition chromatography (CPC), a batch process for time‐dependent separation of complex molecules with the help of a two‐phase system [20, 21]. In addition to the batch process, there are various approaches for implementation as a continuous process, such as the multiple dual‐mode CPC [22], the sequential CPC [23], or the intermittent counter‐current extraction [24]. CPC is applicable, for example, for the purification of natural substances, enantiomeric separation, or purification of biomolecules such as antibodies [25, 26, 27]. The same applies to the separations planned for CPE [19].

schwienheer presented a first promising proof of concept regarding the fluid flow in the apparatus [19]. The unique feature of the CPE concept is the creation of a quasicontinuous multistage partitioning process via a counter‐current flow of two immiscible phases taking place in the centrifugal field of a single rotating device. Analogous to CPC, the central part is a rotor with cascaded chambers.

**FIGURE 1:**
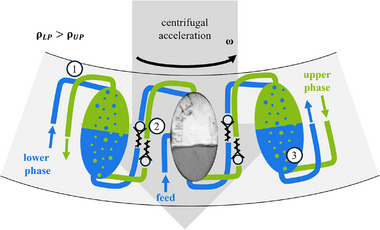
Working principle of the CPE based on three representative chambers as an example for the overall setup. With the challenges described by schwienheer: (1) crossing ducts, (2) check valves for a defined flow direction, and (3) a defined flow direction in the end chambers. In the bisecting chamber, a picture of the flow pattern of Arizona‐N in CPE at a volume flow of 20 mL · min^−1^ and a rotational speed of 600 rpm is shown.

In CPE, all chambers are connected in series with two ducts, one for the lower phase and one for the upper phase. The goal is to enable a counter‐current flow of the two phases. The feed is introduced to the process in the bisecting chamber of the rotor. The feed consists of a carrier material (e.g., one of the two immiscible phases) that contains the components to be purified. The counter‐current flow through the chambers enables a large concentration gradient between feed and solvent, analogous to classical extraction processes, while simultaneously eluting the components to be separated at the two different outlets [18, 19]. The schematic setup of CPE is shown in Figure 1.

schwienheer described three challenges with potential solution concepts in his work [18, 19]: Crossing ducts (1), undefined flow direction inside the ducts (2), and bypass streams inside the end chambers of the cascade (3). Admitting crossing ducts and defining a concrete flow path for each phase, the rotor was developed, consisting of three plates and several check valves. Inside the stacked rotor plates, the counter‐current flow of the two liquid phases through separate ducts was realized. As a result, the two phases contact inside the chambers only. Accordingly, only one of the two phases flows through the ducts. Check valves define the flow direction in each duct in the system and thus the passing phase. Additionally, bypasses from the first separation chamber directly into the outlet of the other phase are avoided by using high‐speed valves in the periphery of the system, resulting in an alternating flow.

The counter‐current flow in CPE is based on the combination of the two operation modes adapted from CPC. First, the so‐called ascending mode, where the upper phase is pumped as the mobile phase through the rotor and the lower phase functions as the stationary phase. The second mode is the so‐called descending mode. Here, the lower phase is the mobile phase while the upper phase remains stationary. The alternated flow direction with switches between ascending and descending mode results in a pseudocontinuous counter‐current flow. Further, the shorter the sequence time, the more accurate the approximation of a continuous operation mode [18, 28].

Substances to be separated can be fed to the process during the ascending phase or during the descending phase, and elute time dependently based on their affinity to the mobile and the stationary phase [18, 28, 29].

Due to the chamber design, the mobile phase disperses as it enters the chamber and coalesces at the opposite side, because of the centrifugal accelerations and the unsteady widening from the duct into the chambers. The density differences of the two phases enable a flow in opposite directions through the chambers. The lower phase, with its higher density compared with the upper phase, flows in centrifugal direction, while the upper phase flows in centripetal direction. Based on this behavior, the chamber can be divided into a dispersion zone and a coalescence zone. The location of the two zones switches analogous to the operating mode. In the radial direction, the dispersion zone is at the outer side in ascending mode and at the inner side in descending mode.

The efficiency of the separation stages is mainly influenced by the interfacial area. The larger the interfacial area, the higher the mass transfer. The interfacial area itself is influenced by the operating parameters, flow rate of the respective liquid, and rotational speed, as well as by the design parameters like shape and arrangement of chambers and ducts [19, 30, 31, 32].

For an optimal separation process, the finest possible dispersion for a fast phase partition is desired. Here, the degree of dispersion also depends on the operating parameters, the selected phase system, as well as the design of the chambers and ducts.

Related to the hold‐up of the stationary phase, there are some important differences between CPC and CPE. While the stationary phase holdup should be as large as possible in CPC [32, 33], the volume fraction of the lower and upper phase in CPE is aimed to be equal. As a continuous process, the operation of CPE has to be in a steady state, which means that the mass transfer of the target components between the two phases should also be stationary during the entire processing time. This can be ensured by keeping the mass transfer area and the accessible volume of the two phases constant [19].

The accessible volume of the two phases can be described by the phase ratio (Equation 1).

(1) $$\alpha_{\mathrm{UP}}=V_{\mathrm{UP}}\;\cdot {V_{\mathrm{total}}}^{-1}.$$

The phase ratio $\alpha_{\mathrm{UP}}$ is defined as the volume of the upper phase $V_{\mathrm{UP}}$ in relation to the total rotor volume $V_{\mathrm{total}}$: As the chambers are completely filled with the two liquid phases, the sum of $\alpha_{\mathrm{UP}}$ and $\alpha_{\mathrm{LP}}$ in every chamber equals 1.

The schwienheer‐based design of the CPE chambers is an initial trial, and the geometry has not been further optimized in terms of hydrodynamics and the associated separation efficiency [18]. In addition, the stacked rotor is sensitive to blockages and leakages at the same time. The numerous nonreturn valves are located inside the rotor and lie, therefore, in the centrifugal field. This means that the rotational forces also act on the locking mechanism and ensure that the balls used for sealing do not run optimally through their seat, and often, not all valves can be opened as required. At the same time, leakages have been observed inside the rotor, which can also be caused by the effect of the rotational forces on the closing mechanism of the nonreturn valves. Further, the CPE process is performed with alternating pump direction, so the ducts of the lower phase and the upper phase are never flowing through simultaneously, and the fast switches in the pump direction also lead to potential blocking of the valves [18].

All these points motivate the implementation of an adapted concept called counter‐current centrifugal extraction (CCE). The concept follows the idea of only one duct as a connection between the chambers. Therefore, the basic geometry of CPC rotors is combined with the alternating flow direction and feeding known from CPE. The hypothesis is that the small hold‐up in the ducts does not disturb dispersion and coalescence inside the chambers.

With this, the basic periphery setup of the CPE can be directly transferred to realize the CCE concept; only the rotor design needs to be adapted. Three requirements must be fulfilled to achieve a feasible process with high efficiency.

1. It is mandatory that the chamber volume be significantly larger than the duct volume $V_{\mathrm{duct}}\ll V_{\mathrm{chamber}}$. So, it is ensured that the chambers are not flooded when switching the pump direction. The switch from ascending to descending mode results in the holdup from the ducts $V_{\mathrm{duct}}$ pumped back into the previous chamber, resulting in a change of the phase ratio α in the respective chamber. This can be tolerated when the duct volume is neglectable small compared with the chamber volume.
2. The creation of an interfacial area as large as possible by intensified dispersion of the two phases must be realized based on the overall size and geometry of the chambers. At the same time, it is necessary that a coalescence zone forms for both phases. This requirement has to be fulfilled for a wide range of flow velocities and rotational speeds.
3. The chambers have to be as small as possible to ensure the implementation of a high number of chambers on one rotor. In perspective, several rotors can be stacked to provide a flexible number of separation stages in a compact design.

This work, based on a CFD simulation, aims to demonstrate that the described concept of CCE can be implemented on a hydrodynamic level. Consequently, it needs to be shown that the phase ratio $\alpha_{\mathrm{UP}}$ is kept constant during alternating operation, and an efficient dispersion over the entire chamber volume can be realized by a new chamber design of CCE.

## 2 Materials and Methods

### 2.1 Computational Fluid Dynamics

The most important steps in the implementation of a CFD model are the setup of the process‐specific parameters combined with the initial and boundary conditions, as well as the selection of the solution methods and the computational mesh. These parameters are interdependent and determine the computational effort and the accuracy of the solution.

Balance equations form the basis of the mathematical model of CFD simulations. Based on the three conservation equations for mass, momentum, and energy, a whole system can be described [34].

Due to a moderate pressure level (p<60bar) and the exclusive presence of liquid phases inside the CCE rotor, the assumption of incompressible fluids can be made [35]. This means that the density of the respective fluid does not depend on the pressure. However, the density is not constant but depends on the phase composition in the respective control volume. In this work, the multiphase system is calculated based on the volume of fluid (VoF) method [36].

Transferring the Euler–Euler‐based VoF to the simulation of the two‐phase flow in CCE, the cambers simulated were divided into a finite number of volume elements, so‐called control volumes (CV). For every CV, the phase ratio $\alpha_{\mathrm{CV}}$ describes the volume of the upper phase in every CV $V_{\mathrm{UP}}$ compared with the total volume of the CV $V_{\mathrm{CV}}$ [36] (Equation 2).

(2) $$\alpha_{\mathrm{CV},\mathrm{UP}}=V_{\mathrm{UP}}\;\cdot {V_{\mathrm{CV}}}^{-1}.$$

Neglecting mass transfer between the two phases, the discretization of the phase fraction α in every CV from timestep n to timestep n+1 over a certain timespan Δt results in Equation (3) [36, 37]:

(3) $$\begin{array}{ccc} & & \left(\alpha_{CV,UP}^{n+1}\cdot \rho_{CV}^{n+1}-\alpha_{CV,UP}^{n}\cdot \rho_{CV}^{n}\right)\cdot V_{CV}\cdot \Delta t^{-1}\\ & & \;+\sum_{f}\;\left(\alpha_{f,UP}^{n+1}\cdot \rho_{CV}^{n+1}\cdot U_{f}^{n+1}\right)=0. \end{array}$$

In sum, the volume flux along the CV faces f is taken into account by the factor $U_{f}^{n+1}$. The mean density and dynamic viscosity of every fluid element can be expressed as a function of the phase ratio regarding the control volume (Equations 4 and 5):

(4) $$\rho_{\mathrm{CV}}=\left(1-\alpha_{\mathrm{CV},\mathrm{UP}}\right)\cdot \rho_{\mathrm{LP}}+\alpha_{\mathrm{CV},\mathrm{UP}}\cdot \rho_{\mathrm{UP}},$$

(5) $$\eta_{\mathrm{CV}}=\left(1-\alpha_{\mathrm{CV},\mathrm{UP}}\right)\cdot \eta_{\mathrm{LP}}+\alpha_{\mathrm{CV},\mathrm{UP}}\cdot \eta_{\mathrm{UP}},$$

Consequently, the continuity equation results (Equation 6):

(6) $$\frac{\partial \rho }{\partial t}+\nabla \cdot \left(\rho \underline{u}\right)=0.$$

Furthermore, the most commonly used and calculated fluids in CCE are Newtonian fluids [35]. Therefore, it can be assumed that the shear stress can be described as a function of the dynamic viscosity (η) [38] (Equation 7):

(7) $$\Delta \;\underline{\tau }=\nabla \cdot \left(\eta \nabla \underline{u}\right)$$

The influence of the interfacial tension was calculated using the continuum surface force model implemented in Ansys Fluent. The model determines the pressure change at the interfacial area using the Young‐Laplace equation. For the two‐phase system simulated in this work, the resulting volume force can be described as a function of the curvature $\kappa_{\mathrm{LP}}$ of the interfacial area [37, 39] (Equation 8):

(8) $$F_{\gamma_{\mathrm{UP},\mathrm{LP}}}=\gamma_{\mathrm{UP},\mathrm{LP}}\cdot \frac{\rho \cdot \kappa_{\mathrm{LP}}\cdot \nabla \alpha_{\mathrm{LP}}}{0.5\cdot \left(\rho_{\mathrm{UP}}+\rho_{\mathrm{LP}}\right)},$$

where ρ describes mixed density determined according to Equation (4), while the surface tension coefficient is set constant for the simulation of the two‐phase system and the operational conditions present. According to the rotational forces, the resulting surface force is implemented into the model as a source term $\left(S_{F}\right)$ [37].

Based on the assumption of isothermal operation, the Navier–Stokes equation can be applied [34, 38] (Equation 9):

(9) $$\begin{array}{c} \frac{\partial \left(\rho \underline{u}\right)}{\partial t}+\nabla \cdot \;\left(\rho \underline{u}\;\underline{u}\right)=-\nabla p+\nabla \cdot \left[\eta \left(\nabla \underline{u}+\nabla {\underline{u}}^{T}\right)\right]+\rho \underline{g}+S_{F}, \end{array}$$

where the gravitational acceleration is indicated by $\underline{g}$. Additional accelerations, in the context of CCE, due to the rotation of the system, are taken into account in the source terms ($S_{F}$). For a coordinate system with its origin located in the centre of the rotation axis at the lower phase of the rotor in combination with a rotating reference system, the centrifugal and Coriolis accelerations have to be considered in the simulation. The centrifugal acceleration (${\underline{a}}_{cf}$) acts orthogonally to the axis of rotation and depends on the angular velocity (${\underline{\omega }}_{a}$), as well as the distance of a fluid element from the axis of rotation ($\underline{r}$) [35] (Equation 10):

(10) $${\underline{a}}_{cf}={\underline{\omega }}_{a}\times \left({\underline{\omega }}_{a}\times \underline{r}\right).$$

The Coriolis acceleration depends on the velocity vectors ($\underline{u}$) at a certain time and is always orthogonal to them [35, 40] (Equation 11):

(11) $${\underline{a}}_{Co}=-2\cdot {\underline{\omega }}_{a}\times \underline{u}.$$

The sum of the rotational accelerations ensures that a fluid element is not moved outwards in a straight line, but along a spiral line depending on its initial speed. These equations enable the fluid movement in CCE to be modelled, but model‐specific boundary and initial conditions are required to solve the CFD simulations. The boundaries of the CCE will be divided into top, bottom, wall, inlet_LP, inlet_UP, outlet_LP, and outlet_UP,  based on the physical rotor.

Top, bottom, and wall are the mechanical boundaries of the chambers and therefore defined as static walls with the help of the no‐slip boundary condition (Equation 12):

(12) $${\underline{u}}_{top}={\underline{u}}_{bottom}={\underline{u}}_{wall}=0.$$

Furthermore, the contact angle between the wall and the two‐phase system has a strong influence on the curvature of the surface. Hence, different contact angles $\theta_{\mathrm{contact}}$, dependent on the surface material, and the two‐phase system was implemented. In CPE top and bottom were sealed with FEP foil, and the wall material is stainless steel. The contact angle between FEP foil and the two Arizona N phases is 127.8 ± 3.4° [41], and the one between Arizona N and stainless steel is 121.9 ± 5.7° [41].

The CCE prototype is produced by additive manufacturing with High Temp T2 V2 resin. Here, the contact angle between Arizona N and all walls is 115.7 ± 3.9°.

The inlets of the respective chambers are defined based on the velocity of the inflowing fluid $v_{\mathrm{in}}$ and the outlets on their pressure. The most important aspect of CCE simulation is the characteristic sequential switching. Accordingly, the boundary conditions vary depending on the switching sequence and flow direction. The flow through the inlet, so‐called active inlet, is defined by the velocity and the phase ratio of the inflowing fluid as an inlet boundary condition, while the outlet depends on the pressure of the outflowing fluid. The inactive inlet and outlet are described based on the static wall conditions at the same time. After a specific time interval, the passive and active inlets are exchanged, corresponding to the flow mode from descending to ascending and vice versa. This time interval is called sequence time. The code implemented is available at TUDoData https://doi.org/10.17877/TUDODATA‐2025‐MBJHTLDP.

The solution algorithm for the VoF method utilized in this study is implemented in the CFD software Ansys Fluent [37]. In this context, numerous additional implemented algorithms were combined to calculate the interfacial area. The piecewise linear interface construction (PLIC) algorithm was employed since it provides the most accurate description of its course and size. The discretization of the pressure was performed utilizing the PRESTO! An algorithm, which is based on the SIMPLE scheme and recommended for pressure interpolation in the context of the VoF method. The turbulence in the chambers was considered employing the k‐ω‐SST model, enabling a valid description of the fluid dynamics near the wall and in the core of the chamber [34, 37].

Considering the main assumption of momentum conservation, the gradients were determined by the least square cell‐based method connected to the finite volume method in the central node of each cell. Moreover, the convective term was specified iteratively via the second‐order upwind method. The transient discretization was solved via the first‐order implicit Euler approach [37].

A calculation mesh was utilized to divide the total flow area into a finite number of volume cells as required. The mesh influences the computational effort and the quality of the solution significantly. In principle, the finer the mesh, the more accurate the simulation results, but also the higher the computing effort. Thus, a trade‐off has been made. The optimal mesh for the flow geometry was determined via a mesh independence study. The phase ratio concerning the upper phase was chosen as a characteristic variable. Furthermore, structured, partially structured, and unstructured meshes were investigated. An initial guess for mesh size was provided by CFD simulations of CPC chambers [35, 41].

The mesh independence study resulted in a calculation mesh for the original CPE chamber divided into 82×10^4^ cells. All further geometries were calculated using analogous mesh sizes (see Supporting Information Material S1)

The CFD simulations were performed using Ansys 2022R2 [37]. The software includes a mesh generation tool (Ansys MESHING), the solver Ansys Fluent, and the evaluation program Ansys CFD‐Post [37]. The simulations were carried out on the Linux high‐performance computing cluster LiDo3 at TU Dortmund University.

### 2.2 Transfer of Operating Parameter and Phase System into the CFD Simulation

In this work, the Arizona N system is used as a model system. It consists of equal volumetric amounts of ethyl acetate, n‐heptane, methanol, and water and was also used for several CPC characterizations successfully [19, 42].

The physical properties of the Arizona N system implemented in the simulation model include density ($\rho_{\mathrm{UP}}=$ 784.4 ± 2.2 kg∙m^−3^, $\rho_{\mathrm{LP}}=$ 928.1 ± 10.8 kg∙m^−3^), dynamic viscosity ($\eta_{\mathrm{UP}}=$0.3755 ± 0.0017 mPas, $\eta_{\mathrm{LP}}=$ 1.462 ± 0.0047 mPas), and surface tension between the two liquid phases and the wall materials of the rotor ($\gamma_{\mathrm{UP},\mathrm{LP},\mathrm{wall}}=$ 2.9717 ± 0.1776) [43].

Getting an overview of the feasibility of the operation concept of CPE and CCE, moderate operating parameters were considered. The rotational speed was set to 600 rpm, and volumetric flow rates of 20 and 40 mL · min^−1^ were simulated. The last operating parameter is the sequence time, which was set to 1 s in the simulations. As a result, it is possible to investigate several switches in the simulation. With this setting, the simulation differs from realistic experimental values, where the minimum sequence time is 5 s up to 90 s. Due to the stable hydrodynamics over time, the short sequence times are also representative of the qualitative behavior in the experiments.

The contact angles and properties of the Arizona N system refer to the two‐phase system made of equal volume fractions of ultrapure water (Milli‐Q Advantage A10, 0.05 µS cm^−1^ Merck KgaA, Darmstadt), methanol (99.8%, VWR Chemicals, Radnor, PA, USA), ethyl acetate (99.8%, VWR Chemicals), and n‐heptane (99.8%, VWR Chemicals). For a comparison of the fluid dynamics inside the CPE chambers, 4 mg · L^−1^ methylene blue (100%, VWR Chemicals) was added to dye the heavy, aqueous, lower phase to visualize the flow regime. fromme has shown that dyeing with methylene blue does not affect the material and fluid characteristics [43].

## 3 Results

### 3.1 Validation of the Basic Model

**FIGURE 2:**
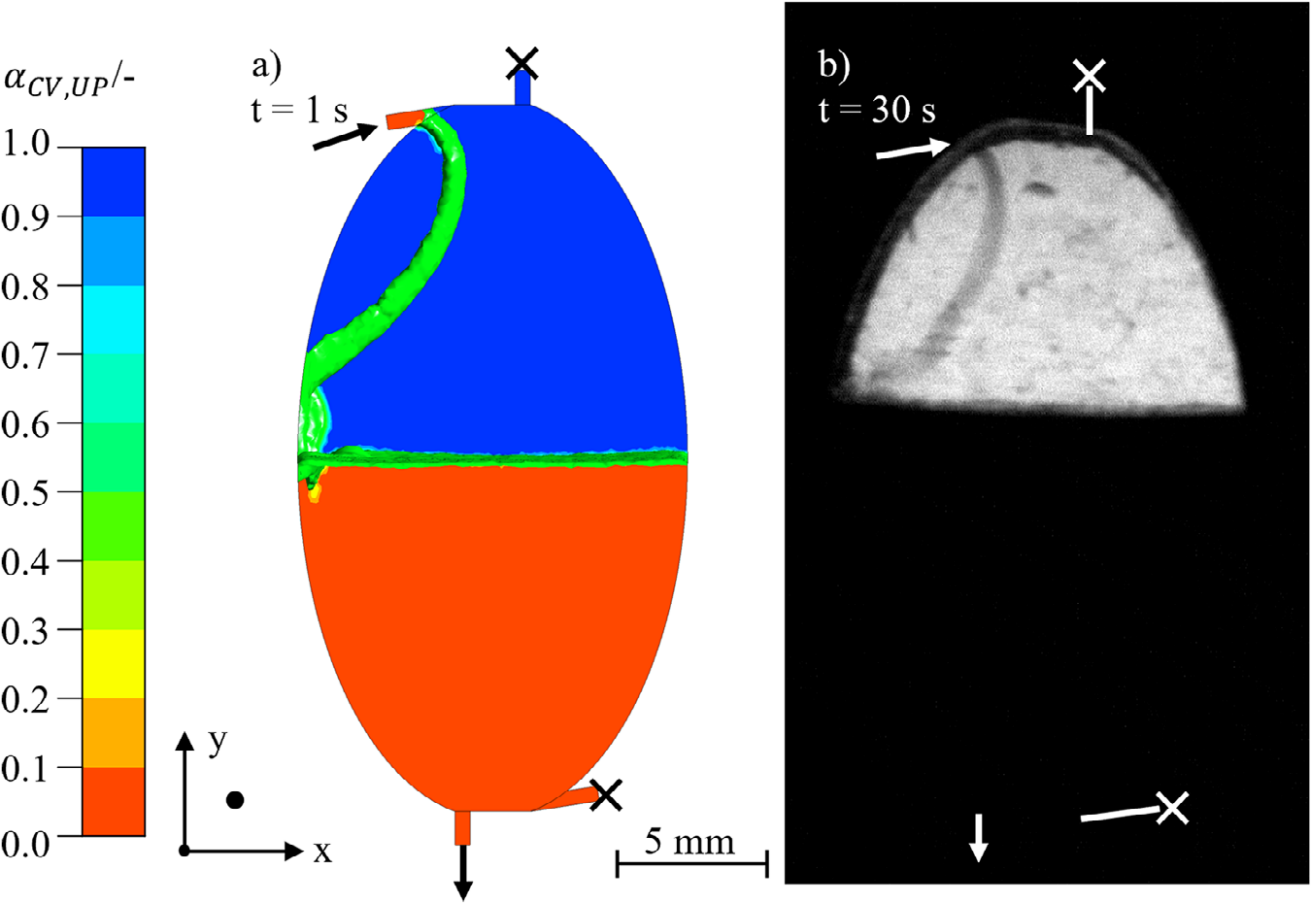
Comparison of simulated (A) and experimental (B) results. Both pictures were taken at 20 mL · min^−1^ at 600 rpm with the Arizona N system in descending mode. The lower phase is dyed with 10 mg L^−1^ methylene blue for a better contrast in the experiments. The arrows mark the flow direction of the inlets and outlets, the crosses mark the inlets and outlets not flown through in descending mode.

A comparison of the flow pattern during the mesh independence study with flow patterns from the inside of the rotor supports the thesis that the selected computational mesh represents a sufficient level of detail. For comparison, the simulated phase ratio in the yx‐plane was compared with the recorded images under the same operating conditions. The focus of the comparison here is primarily on the position of the phase boundary, but also on the observation of turbulence and dead zones. The validation of the simulated results from CPE was performed qualitatively using camera‐based online analytics of hydrodynamics. The CPE setup is described in detail in the work of schwienheer [ 19 ]. The simulated data are shown in Figure 2A and compared with the experimental results in Figure 2B.

Since in the simulation an observation in all directions and all layers is possible, the experimental results are limited to an observation from top to bottom of the chambers. This point of view corresponds to the xy‐plane in the simulation. To ensure a valid comparison, a layer at the position of 10 mm in the z‐direction was used for the observation of simulated chambers (overall chamber height 12 mm).

Both pictures show the inflowing lower phase of the Arizona N system in descending mode for a volume flow of 20 mL · min^−1^ and rotational speed of 600 rpm. At first glance, it is obvious that the phase ratio of the upper phase in the simulated results is $\alpha_{\mathrm{simulated},\;\mathrm{UP}}=$ 0.5, while the phase ratio in the experimental results is $\alpha_{\mathrm{experiemental},\;\mathrm{UP}}=$ 0.45. This effect is caused by the instability of the coalescence zone due to a nonoptimal chamber design. In the simulated results, the coalescence zone is built up after 1 s, so that the phase ratio in the chamber is equal to the phase ratio for the initial conditions of the simulation (see Section 2.1). For the experiments, it is more complex to reach an initial phase ratio of $\alpha_{\mathrm{experiemental},\;\mathrm{UP}}=$ 0.5, and further, the pictures from the experiments were taken after switching from ascending to descending mode with a run time of t= 30 s. During this time, the upper phase is flushed out of the chambers (bleeding, also known as CPC [33]). The elapsed time in the experimental images cannot be avoided, as the apparatus has to be started in ascending mode, and the preparation of the image acquisition requires a corresponding amount of time.

In a further evaluation of the simulated results, not only the influence of the implemented physical parameters but also the selected numerical models must be taken into account. This is especially relevant when examining the dispersion behavior of the inflowing phase as it enters the chambers through the unsteady widening. With an element size of 0.2 mm in the calculation mesh, the simulation is restricted to droplets of comparatively large diameters. This limitation is further influenced by the application of the Continuum Surface Force model for the representation of interfacial tension. As the model determines the interfacial forces based on the curvature of the interface, the accurate resolution of sharp edges (≤90°) is limited. A smaller mesh size and the use of the continuum surface stress model can overcome these limitations, but would increase the computational effort enormously (half element size results in doubled element number). Consequently, the level of detail in the simulations was assessed with a focus on demonstrating the feasibility of the CCE concept [35, 37, 44].

Therefore, when comparing the flow patterns, the main focus should be on the position and shape of the inflowing lamella of the lower phase. The lamella has a semielliptical shape and hits the wall at exactly the same position in both images. This characteristic is due to the Coriolis force [35, 40]. The images show that the simulated results reproduce the real flow pattern very well, so that an optimization of the chamber design is possible based on the simulation results.

### 3.2 Development of CCE Chambers

The CPE duct path, referred to as two‐duct‐connection, was designed for true continuous operation, where both phases flow simultaneously in counter‐current direction [18]. However, this is not possible due to the bypass flows [19]. The setup using check valves is sensitive, and the utilization of the chambers regarding dispersion is <25% of the overall chamber volume. Therefore, the operation mode, including the chambers and ducts, was redesigned and is presented in the following sections.

#### 3.2.1 Chamber Volume and Height

**FIGURE 3:**
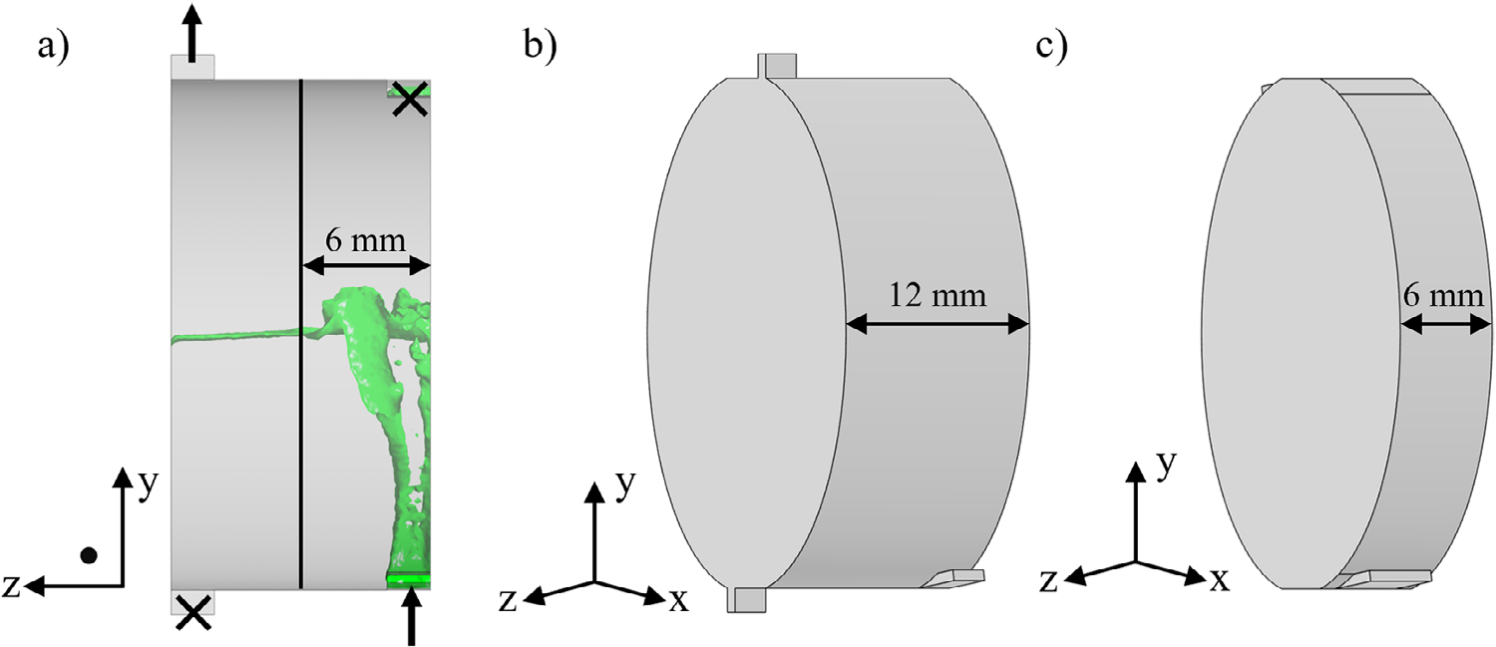
Comparison of the duct paths and chamber heights: (A) side view of the original CPE chamber and the simulated inflowing Arizona N upper phase at 40 mL · min^−1^ and 600 rpm; (B) 3D model of the original CPE chamber; (C) 3D model of the first step in optimization, a CCE chamber with adapted height of 6 mm and ducts of 4 mm.

**FIGURE 4:**
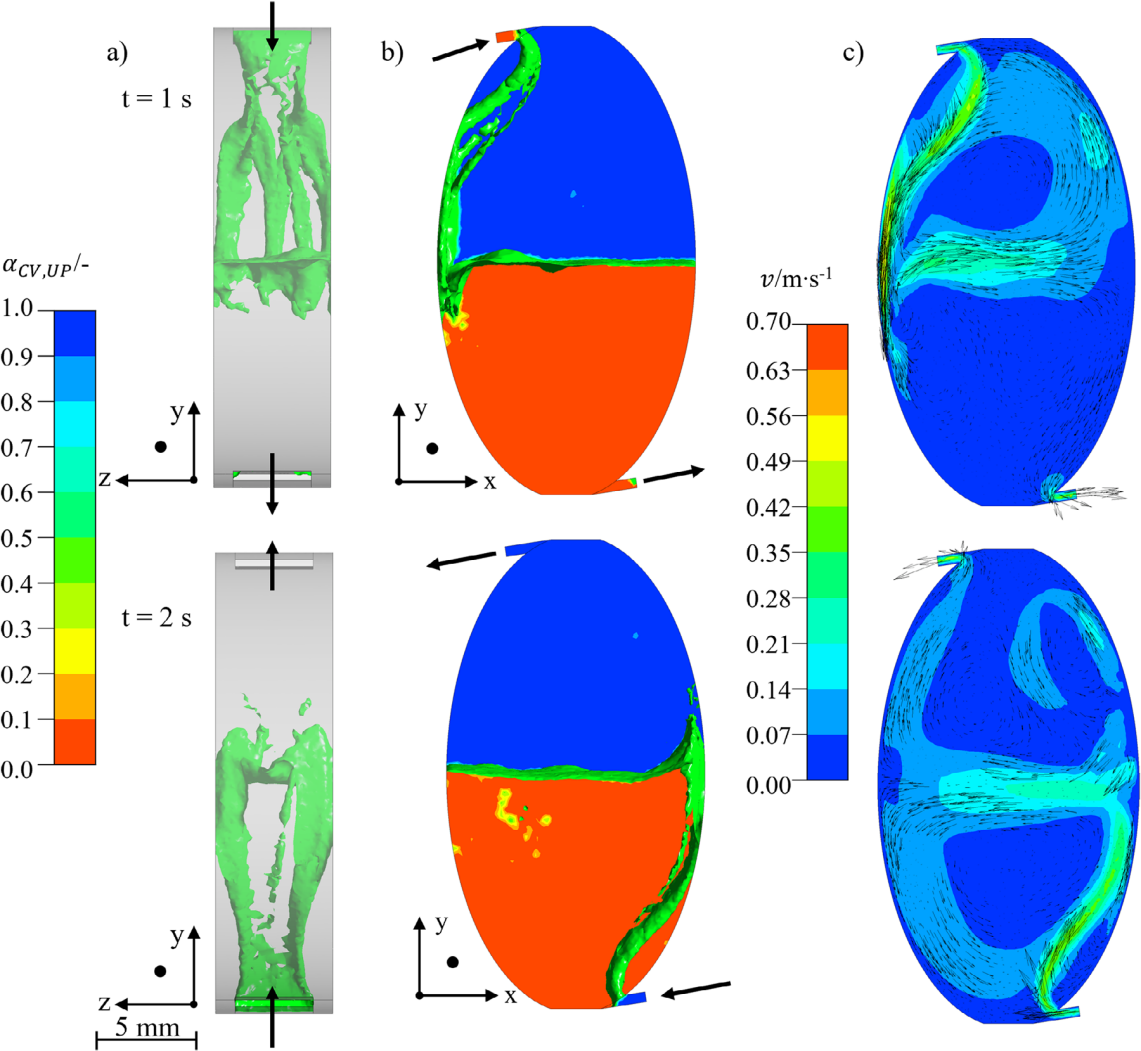
(A, B) Interface and volume fraction in descending (top) and ascending mode (bottom). The interfacial area is indicated by an isosurface at α= 0.5 in green. The UP is shown in blue and the LP in red. (C) Velocity profile represented by contours. The direction of the velocity is shown as vectors, Arizona N with $v_{\mathrm{in}}=$ 40 mL · min^−1^, n= 600 rpm, and $t_{\mathrm{seq}}=$ 1 s. The coordinate direction shows the camera perspective.

**FIGURE 5:**
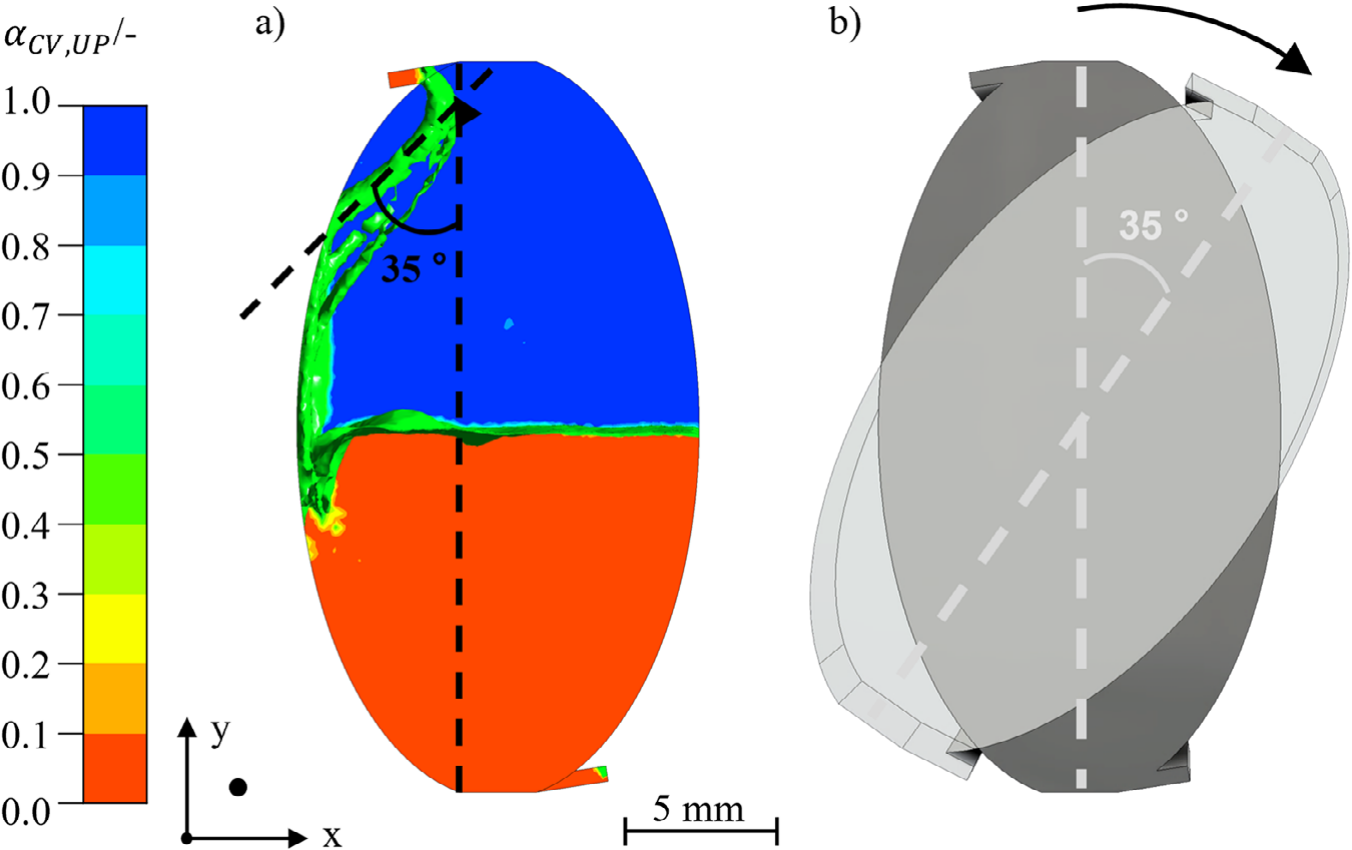
Determination of the inclining angle of the chamber. (A) An example of the determination of the angle of the incoming lamella. The interfacial area is indicated by an isosurface at α= 0.5 in green. The UP is shown in blue and the LP in red. Arizona N with $v_{\mathrm{in}}=$ 40 mL · min^−1^, n= 600 rpm, and $t_{\mathrm{seq}}=$ 1 s. The coordinate direction shows the camera perspective. (B) Schematical inclination of the chamber.

**FIGURE 6:**
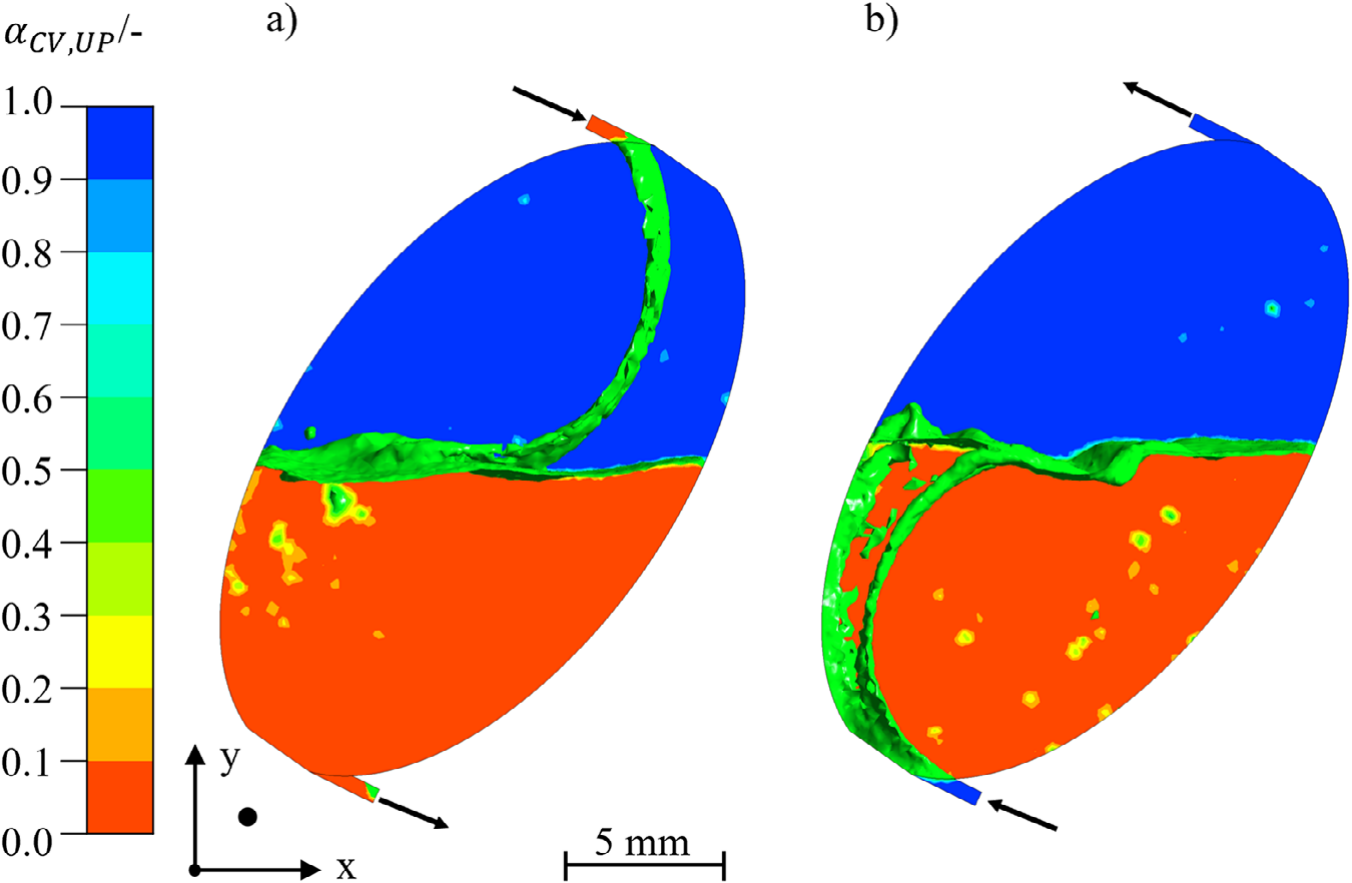
Comparison of the flow patterns for tilt angles of 35°. (A) ascending mode and (B) descending mode. The interfacial area is indicated by an isosurface at α= 0.5 in green. The UP is shown in blue and the LP in red. Arizona N with $v_{\mathrm{in}}=$ 40 mL · min^−1^, n= 600 rpm, and $t_{\mathrm{seq}}=$ 1 s. The coordinate direction describes the camera perspective.

In the first step, instead of chambers connected via two ducts each, a connection of the chambers with one inlet and one outlet per chamber was used. Further, due to the low utilization of the chamber in the z‐direction (see Figure 3A), the chamber height was halved from 12 mm (CPE, Figure 3B) to 6 mm (CCE, Figure 3C). This enables the equipment of twice as many rotor plates in the housing and thus the realization of twice as many theoretical separation stages.

Furthermore, by manufacturing the chambers via additive manufacturing, the position, shape, and size of the inlets are no longer dictated by the rotor plates. Therefore, these are enlarged to provide a larger interfacial area and a uniform flow through the chamber. Since an increased inlet area also results in a lower inlet velocity, a compromise must be found. The inlet height was doubled from 2 mm (CPE) to 4 mm (CCE), and the inlet was positioned in the centre to minimize the wall interaction of the incoming lamella. The described transformed geometry is shown in Figure 3.

Since the inlet speed was kept constant compared with the previous simulations, the optimization steps were carried out at a flow rate of 40 mL · min^−1^. The rotational speed was kept at 600 rpm to ensure comparability. The resulting flow patterns and velocity profiles are shown in Figure 4.

The lamella of the inflowing phase lies close to the chamber wall. This means that the modification did not improve the chamber utilization in the xy‐plane. However, in the z‐direction, the chamber was completely flown through by the lamella. This provides a larger interfacial area and intensified mass transfer compared with the original chamber design. An improved mixing can also be observed by the velocity profiles inside the chambers. A vortex around the entire dispersion zone is visible in the simulations.

This intensified mixing favours convective mass transfer and thus the separation efficiency of the apparatus. Consequently, a smaller chamber improves chamber performance. The utilization of the chambers in the xy‐plane requires more modifications of the chamber design.

The next optimization step is to adjust the chamber orientation to optimize the chamber utilization. By inclining the chamber in the xy‐plane, the deflection of the lamella toward the opposite chamber wall due to the Coriolis force is prevented. The aim is to influence the position of the phase interface based on the chamber design to maximize the mass transfer due to the largest possible interfacial area.

To determine the chamber angle, the angle of the inflowing lamella was determined for different rotating speeds and volume flows. For volume flows from 10 to 40 mL · min^−1^, the angles of the inflowing lamella were between 33° and 38° in both operation modes. The exemplary determination of the chamber angle and a schematic comparison of the not‐inclined chamber are demonstrated in Figure 5A, and the inclined one in Figure 5B.

Concerning the aim of a maximized free‐flowing lamella respective dispersion in the chambers without wall contact, the measured angles are utilized for the orientation of the chambers because the chambers should be tilted parallel to the lamellas. With a mean angle of 35° for the lamella, a chamber design with the same angle was implemented in the simulations.

#### 3.2.2 Chamber and Duct Orientation

Based on the results of Section 3.2.1, a chamber with 35° rotation was simulated using analogous operating conditions with the goal of reaching a free‐flowing mobile phase through the dispersion zone. The resulting flow pattern is depicted in Figure 6.

For the descending mode, the requirement of a free‐flowing lower phase is fulfilled (see Figure 6A), but in the ascending mode, the tilting of the chamber results in the lamella not touching the left wall, as without tilting, but the right wall (see Figure 6B). This effect is the result of different densities of the phases and different resulting Coriolis forces combined with the opposing inflow directions [40]. As the upper phase flowing in the ascending direction has a lower density, the Coriolis force is no longer sufficient to accelerate the fluid elements in the direction of the right chamber wall, so that the inflowing lamella clings to the left chamber wall (see Equation 11).

This effect can be counteracted by directing the incoming phase in a corresponding direction as it enters the chamber. This is because the Coriolis force is a function of the velocity and its direction, resulting in a particular influence of the inlet angle. The stainless‐steel chamber has an inlet angle of −8.3°. To force the flow of the incoming phase more to the right side in ascending mode, an inflow with a more acute angle of incidence is recommended. To investigate the influence of this angle, inlet angles of 10°, 20°, 30°, 40°, and 55° were compared.

**FIGURE 7:**
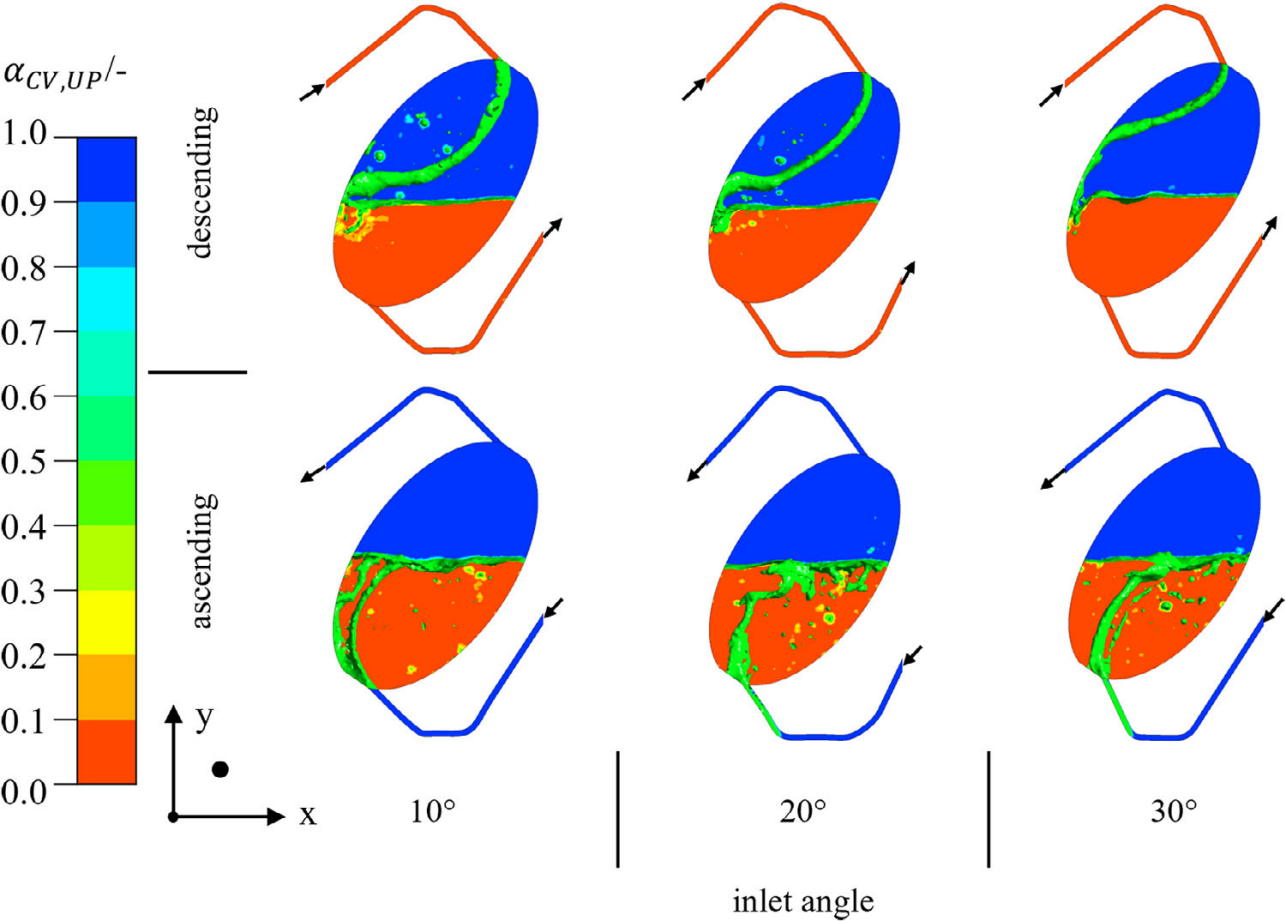
Comparison of the flow scheme in chambers oriented at 35° with various inlet angles. The interfacial area is indicated by an isosurface at α= 0.5 in green. The UP is shown in blue and the LPs in red. Arizona N with $v_{\mathrm{in}}=$ 40 mL · min^−1^, n= 600 rpm, and $t_{\mathrm{seq}}=$ 1 s. The coordinate direction shows the camera perspective.

The change in the inlet angle is intended to prevent the lamella from adhering to the wall. Especially in ascending mode, a flow through the chamber is to be facilitated centrally. The flow schemes for the different inlet angles are shown in Figure 7.

Depending on the inlet angle, the radius of the flow path of the incoming lamella changes. The larger the angle, the smaller the radius. This can be explained by the direction of action of the inertial force and the Coriolis force that depends on it. The inertial force acts in the direction of the inlet, and the Coriolis force acts normal to it. Therefore, a larger inlet angle leads to a stronger deflection of the lamella [40].

In ascending mode, the lamella does not contact the opposite wall for an inlet angle greater than 10°. Hence, the aimed optimization can be achieved by modifying the angle to 20°. A larger inlet angle further favours the flow of LP into the duct, since the centrifugal force acts strongly in the direction of the inlet. Combined with the ducts, connecting the chambers, siphon effects, and dead zones can appear. Therefore, for an inlet angle of 20°, a free‐flowing inlet phase is present for both operating modes, and no siphons in the ducts appear in descending mode. All investigations on the flow pattern have been done at a moderate volume flow (20–40 mL · min^−1^) and low rotational speed (600 rpm).

In the last step, the focus is on the creation of a more robust chamber design to realize the desired flow behavior in several conceivable combinations of operating conditions.

#### 3.2.3 Baffles for Directed Flow

The aim of chamber optimization is not only to optimize the hydrodynamics for one operating point, but to achieve the most uniform flow behavior possible for different operating parameters. This allows for setting the optimum operating point depending on the particular separation task and not the apparatus. By using baffles, a uniform course of the lamella for different volume flows and rotational speeds might be achieved. In addition, the baffle should stabilize the coalescence zone and prevent the lamella from constricting. The resulting chambers with the baffles are illustrated in the Supporting Information Material S2.

**FIGURE 8:**
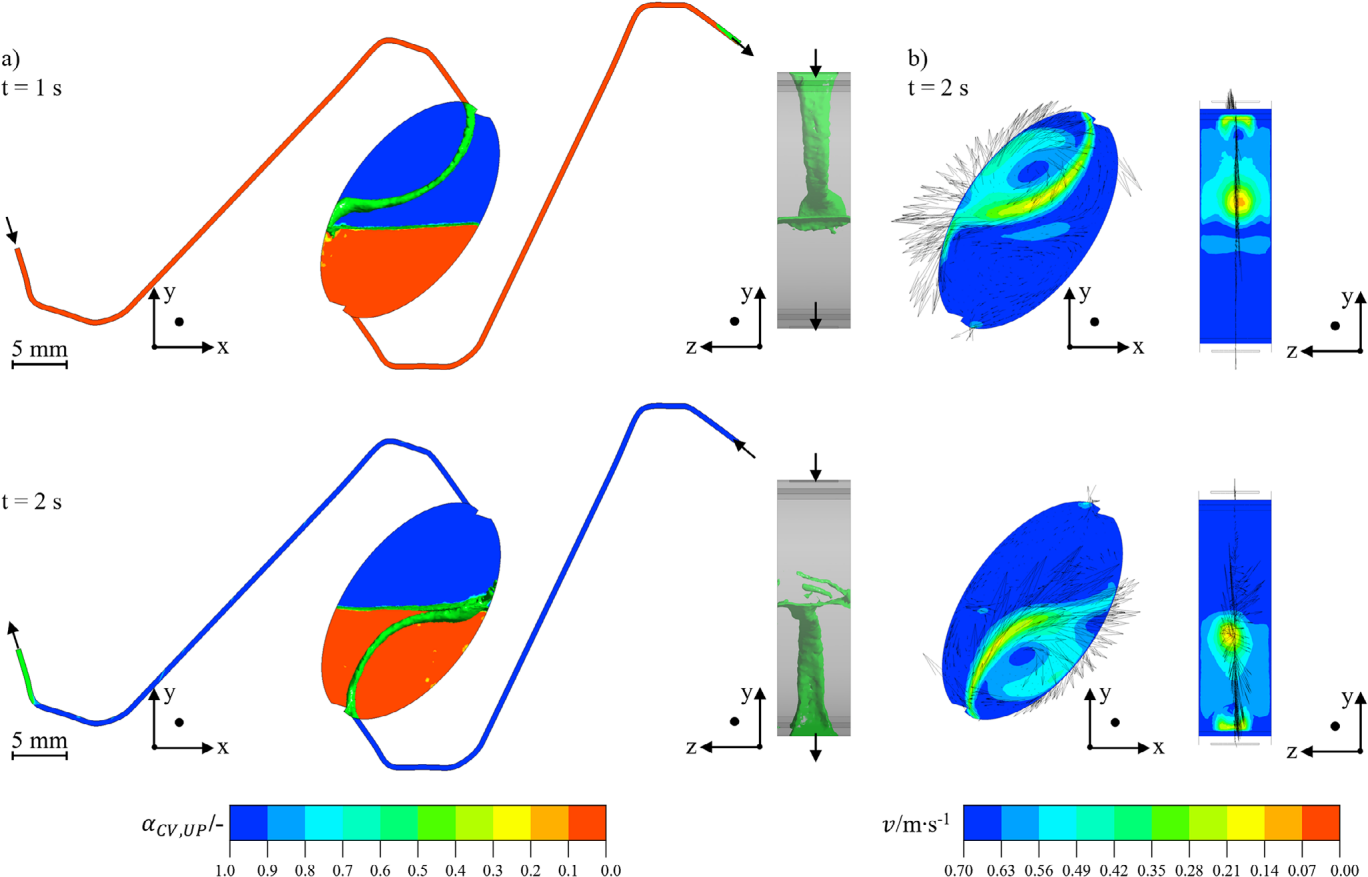
(A) Flow patterns for a chamber oriented at 35° with baffles and an inlet angle of 20° in descending mode (top) and ascending mode (bottom). The interfacial area is indicated by an isosurface at α= 0.5 in green. The UP is shown in blue and the LP in red. (B) Velocity profiles represented by contours and their direction shown as vectors. Arizona N with $v_{\mathrm{in}}=$ 40 mL · min^−1^, n= 600 rpm, and $t_{\mathrm{seq}}=$ 1 s. The coordinate direction describes the camera perspective.

The baffles reach over the full height of the chamber and are 0.5 mm wide, which corresponds to the width of the duct. The distance between the inlet and the baffle is 1 mm to generate a lamella that flows as centrally as possible. Since the chamber is flattened over a length of 2.5 mm at the top and bottom, and this length is to be used completely, the length of the baffles is 1.5 mm. The flow pattern and velocity profiles of the chamber with baffles are depicted in Figure 8.

The baffles ensure that the chamber is flown through centrally. Furthermore, both phases flow approximately mirror‐symmetrically. This becomes evident by the velocity profiles of the chamber that enable an estimation of the mixing quality inside the chamber qualitatively.

In descending mode, a vortex can be seen in the entire upper half of the chamber. This is formed by the inflow in connection with the rotational forces and provides mixing inside the chamber and thus an intensified mass transport. No turbulence can be seen in the lower half of the chamber. Here, the velocities to the outlet are lower compared with the inlet velocity. This behavior indicates efficient coalescence and thus the prevention of phase entrainment. A comparable behavior was identified in ascending mode. Compared with the initial chamber design, the dead zones are reduced. Moreover, the interfacial area is increased because the inflowing phase utilizes the overall chamber volume. All in all, the baffles provide a uniform flow behavior and stabilize the hydrodynamics. Enhanced mixing is achieved at the interfacial area, favouring mass transport.

Finally, the results are elliptical chambers inclined at 35° to the radial direction, with a height of 6 mm and a volume of 1.43 mL. Additionally, baffles are placed inside the chambers opposite to the two inlets, guiding the inflowing phase to the chamber center. With a rectangular cross‐sectional area (B: 0.5 mm, H: 4 mm), the ducts have a volume of 0.17 mL. With this, the final geometry of the chambers and ducts was set. A comparison of the initial CPE chamber geometry and the final CCE geometry can be found in the Supporting Information Material S3.

**FIGURE 9:**
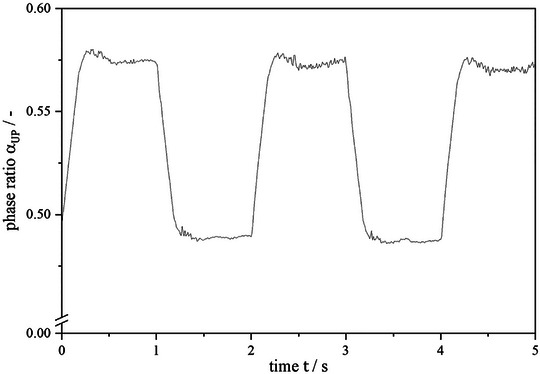
Simulation of phase fraction in a CCE chamber over time. The volumetric flow rate is 40 mL · min^−1^, the rotational speed is 600 rpm, and the sequence time is 1 s. The phase system used is Arizona N.

As the last step, the phase ratio in the chamber was observed over 5 s simulated time to validate whether the phase ratio stays constant despite a single duct connection and the flow of both phases through one duct. In Figure 9, it can be seen that the phase ratio in the chamber is set to 0.5 in the beginning and alternates during the 5 s of operation.

It is striking that the set phase ratio of 0.5 only exists at the start of the simulation and then increases immediately. This effect is caused by the upcoming lamella of the inflowing phase. Starting the simulation in descending mode, the upper phase content in the ducts at *t* = 0 s flows into the chamber, causing an increase in the phase ratio in the chambers. After an overshoot up to 0.58, the phase ratio stabilizes around 0.57 for the ascending mode. At *t* = 1 s the operation mode is switched from ascending to descending. At this point, the lower phase inside the outlet duct is flushed back into the chamber, causing small disturbances at first, resulting in a phase ratio around 0.48. The same is visible in all following switches, with the values of 0.57 in ascending and 0.48 in descending being maintained in each case. This confirms the hypothesis that a counter‐current flow is possible with single ducts connecting the chambers in a CCE rotor. The phase ratio inside the chambers is shifted due to the alternated flow directions, but the disturbance on the hydrodynamics is small enough that the initial stable state is regenerated with every switch during the alternated operation.

## 4 Conclusion and Outlook

In this study, a CFD model was developed to simulate the hydrodynamics inside CPE chambers. Based on this model, a novel operating concept, the so‐called CCE, was developed with the hypothesis that one single duct between the separation chambers is sufficient to create a counter‐current flow. An optimized chamber design was determined with the objective of improving mixing for efficient mass transfer and to achieve hydrodynamically stable behavior, enabling long‐term operation of the plant.

The concept for CCE resulted in a change in the duct design, a reduction in chamber height to 6 mm compared with the initial height of 12 mm in CPE. Due to the Coriolis force acting on the liquid phases, the chambers were tilted 35° to their central axis. In addition, the inlet angle was adjusted to 20°, and baffles were introduced. A simulation of five switches has shown that the phase ratio inside the chambers alternates around the initial value of 0.5. This provided the basis for the flexible operation of the CCE.

In our future work, the new concept and chamber design will be analyzed in a detailed hydrodynamic study, which will show whether the optimized hydrodynamics have been achieved independently of the operating conditions and the phase system used. Further, the simulated geometry will be extended to the overall rotor. The proof of concept will be expanded to experimental studies. Here feasibility of the chamber design optimization will be tested through experimental investigations with a focus on hydrodynamics. Therefore, an additive‐manufactured modular rotor design is appropriate to test different chamber designs and configurations.

Once optimized, the first separation tests will provide the basis for realizing separation processes in the CCE with the potential to generate an efficient multistage extraction process with a small footprint.

## Author Contributions

**Sophia Volpert**: writing – original draft, writing – review & editing, conceptualization, methodology, investigation, software, formal analysis, visualization. **Lisa Nordhausen**: investigation, software, formal analysis, visualization. **Richard Alfsmann**: software, visualization. **Gerhard Schembecker**: writing – review & editing, conceptualization, validation, supervision.

## Conflicts of Interest

The authors declare no conflicts of interest.

## Supporting information




**Supporting File 1**: jssc70339‐sup‐0001‐SuppMat.docx.

## Data Availability

The data that support the findings of this study are openly available on TUDodata at https://doi.org/10.17877/TUDODATA‐2025‐MBJHTLDP.
