# Supplementary material for: Development of a Novel Rotor Design for Counter‐Current Centrifugal Extraction Based on Computational Fluid Dynamics Simulations
Source: J Sep Sci. 2025 Dec 22;48(12):e70339. doi: 10.1002/jssc.70339 (PMC12723355; doi:10.1002/jssc.70339)
Supplement: Supplementary file 1 — Supporting File 1: jssc70339‐sup‐0001‐SuppMat.docx. [file JSSC-48-e70339-s001.docx]

**Supplementary Material**

**Development of a Novel Rotor Design for Counter-Current Centrifugal Extraction based on Computational Fluid Dynamics Simulations**

**Sophia Volpert, Lisa Nordhausen, Richard Alfsmann, Gerhard Schembecker***

Laboratory of Plant and Process Design, Department of Biochemical and Chemical Engineering,

TU Dortmund University, 44227 Dortmund, Germany; sophia.volpert@tu-dortmund.de;

lisa.nordhausen@tu-dortmund.de; richard.alfsmann@tu-dortmund.de;

* Correspondence: gerhard.schembecker@tu-dortmund.de; Tel.: +49-(0)231-755-2339

Table of Content

[S.1. Mesh Independence Study 2](#_Toc212120192)

[S.2. CCE Chamber Geometry 4](#_Toc212120193)

[S.3. Comparison of the CPE Chamber and the CCE Chamber 4](#_Toc212120194)

## Mesh Independence Study

A mesh independence study was performed. A mesh structured in the z-direction and unstructured in the xy-plane was gradually reduced in size and then compared to a completely unstructured mesh. Both meshes are shown in Figure S.1.1.


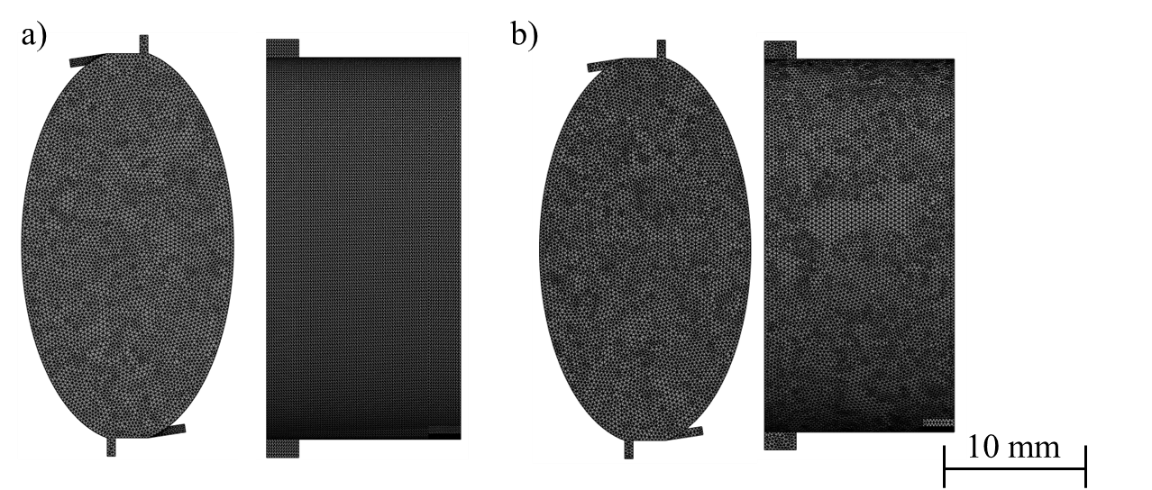


Figure S.1.1: Meshes as visual examples. a) shows the partially structured mesh in xy-plane and z-direction; b) shows the unstructured mesh.

The mesh was first divided into 80 elements in the z-direction. The maximum element size in the xy-plane was set to 0.2 mm. These were then refined individually. An overview of the tested meshes can be found in Table S.1.1.

Table S.1.1: Overview of the characteristics in the mesh independence study.

| l_max_  mm | n_cells_  1∙10^4^ | n_cells,z_  - |  | l_max_  mm | n_cells_  1∙10^4^ | n_cells,z_  - |
| --- | --- | --- | --- | --- | --- | --- |
| 0.2 | 88 | 80 |  | 0.175 | 117 | 80 |
| 0.2 | 99 | 100 |  | 0.150 | 156 | 80 |
| 0.2 | 109 | 120 |  | 0.100 | 335 | 80 |
| 0.2 | 117 | 140 |  |  |  |  |

After the mesh was set all simulations of different flow geometries were performed with analogous mesh size. Using this meshes simulations with several operating conditions, phase systems and geometries, regarding to the main chamber form, the chamber hight and the channel position were set up and analysed.

To find out whether the results are mesh independent, the volume fraction of the upper phase is averaged over the chamber and plotted against the number of elements in the mesh. Since the volume fraction is time dependent, the analysis is performed for different time steps. In addition, a partially structured mesh is compared to a completely unstructured mesh. The results are shown in Figure S.1.2, where the filled symbols represent the partially structured mesh, and the empty ones represent the completely unstructured one.


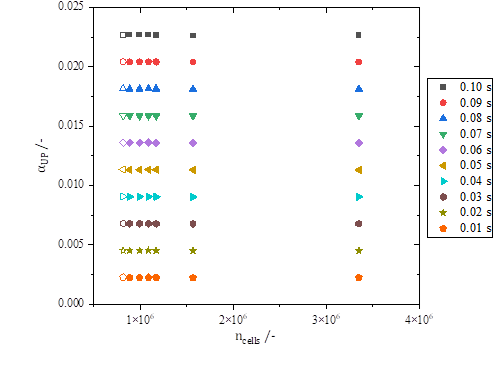


Figure S.1.2: Results of the mesh independence study: The volume fraction of the lower phase versus the number of mesh elements for different time steps. The filled symbols represent the partially structured mesh, and the empty symbols represent the completely unstructured one.

For the meshes considered, the volume fraction is independent of the mesh size. Larger meshes could not be considered because the results for them diverge. Finer meshes were not considered due to the computational complexity and time required for the calculations. The unstructured mesh shows the best convergence behavior, which can be explained by its better adaptability to the geometry. Furthermore, a completely unstructured mesh has the advantage that it can be transferred to different geometries with less effort. Since the optimization of the hydrodynamics in this work shall consider different geometries, the completely unstructured mesh will be used in the further course. This has a maximum element size of 0.2 mm and is shown in Figure S.1.1b). A much finer mesh would allow the representation of fine droplets, but it would have to be finer than most droplets, which would increase the computational effort by about 50 times. Thus, the computational effort would not be in reasonable proportion to the results.

## CCE Chamber Geometry


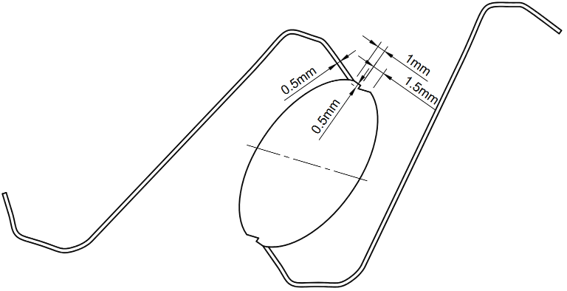


Figure S.2.1: Schematic representation of the chamber equipped with baffles.

## Comparison of the CPE Chamber and the CCE Chamber


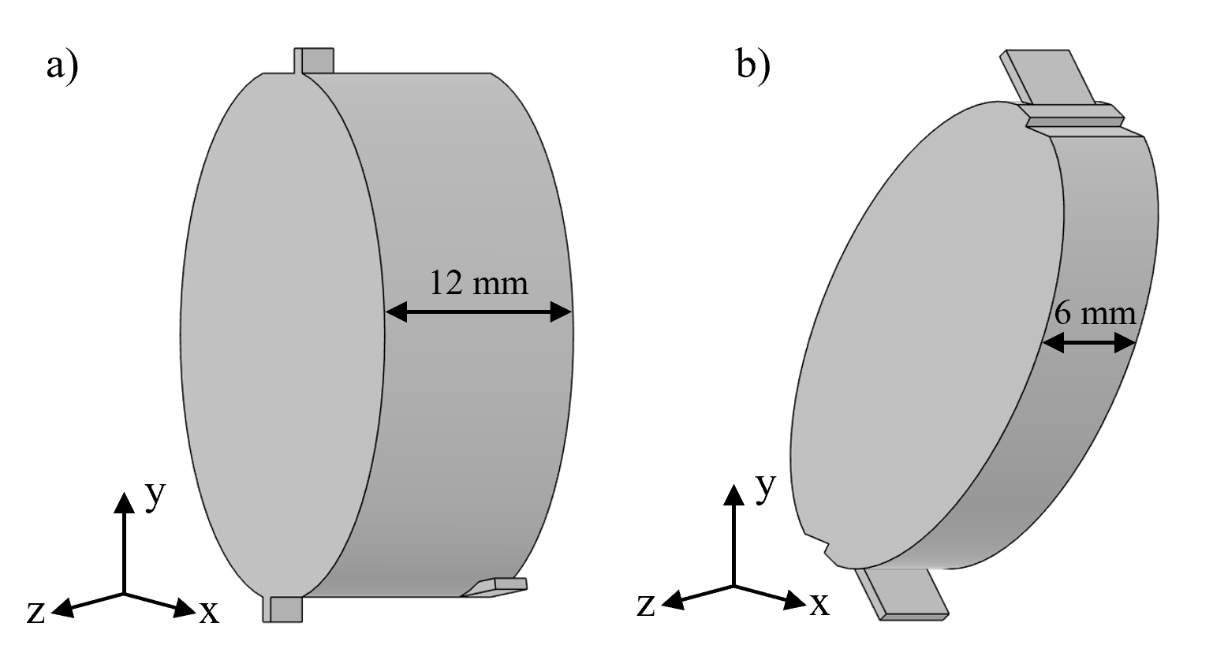


Figure S.2.2: 3D models of the initial CPE flow chamber (a) and the final CCE flow chamber (b).
